# Novel MDM2 Inhibitor XR-2 Exerts Potent Anti-Tumor Efficacy and Overcomes Enzalutamide Resistance in Prostate Cancer

**DOI:** 10.3389/fphar.2022.871259

**Published:** 2022-04-25

**Authors:** Meng Wu, Jingyi Cui, Huimin Hou, Ying Li, Shengjie Liu, Li Wan, Lili Zhang, Wei Huang, Gaoyuan Sun, Jingchao Liu, Pengfei Jin, Shunmin He, Ming Liu

**Affiliations:** ^1^ Department of Urology, Beijing Hospital, National Center of Gerontology, Institute of Geriatric Medicine, Chinese Academy of Medical Sciences, Beijing, China; ^2^ Key Laboratory of RNA Biology, Center for Big Data Research in Health, Institute of Biophysics, Chinese Academy of Sciences, Beijing, China; ^3^ Graduate School of Peking Union Medical College, Beijing, China; ^4^ The Key Laboratory of Geriatrics, Beijing Institute of Geriatrics, Institute of Geriatric Medicine, Chinese Academy of Medical Sciences, Beijing Hospital/National Center of Gerontology of National Health Commission, Beijing, China; ^5^ Clinical Biobank, Beijing Hospital, National Center of Gerontology, National Health Commission, Institute of Geriatric Medicine, Chinese Academy of Medical Sciences, Beijing, China; ^6^ Department of Pharmacy, Beijing Hospital, National Center of Gerontology, Institute of Geriatric Medicine, Chinese Academy of Medical Science, Beijing Key Laboratory of Assessment of Clinical Drugs Risk and Individual Application (Beijing Hospital), Beijing, China

**Keywords:** MDM2 inhibitor, enzalutamide resistance, combination therapy, castration-resistant prostate cancer, p53

## Abstract

**Background:** The inactivation of tumor-suppressor p53 plays an important role in second generation anti-androgens (SGAs) drug resistance and neuroendocrine differentiation in castration-resistant prostate cancer (CRPC). The reactivation of p53 by blocking the MDM2–p53 interaction represents an attractive therapeutic remedy in cancers with wild-type or functional p53. Whether MDM2-p53 inhibitor could overcome SGAs drug resistance in CRPC is still needed further research. Here, we investigated the anti-tumor efficacy and mechanisms of a novel MDM2-p53 inhibitor XR-2 in CRPC.

**Methods:** To investigate the functions and mechanisms of XR-2 in prostate cancer, *in vitro* and *in vivo* biofunctional assays were performed. Western blot and qRT-PCR assay were performed to detect the protein and mRNA expression levels of indicated genes. CCK8, colony formation, flow cytometry and senescence assays were performed for cell function identifications. RNA-sequencing and bioinformatics analysis were mainly used to identify the influence of XR-2 on prostate cancer cells transcriptome. Subcutaneous 22Rv1 derived xenografts mice model was used to investigate the *in vivo* anti-tumor activity of XR-2. In addition, the broad-spectrum anti-tumor activities *in vivo* of XR-2 were evaluated by different xenografts mice models.

**Results:** XR-2 could directly bind to MDM2, potently reactivate the p53 pathway and thus induce cell cycle arrest and apoptosis in wild-type p53 CRPC cell lines. XR-2 also suppresses the AR pathway as p53 regulates AR transcription inhibition and MDM2 participates in AR degradation. As a result, XR-2 efficiently inhibited CRPC cell viability, showed a synergistic effect with enzalutamide and overcame enzalutamide resistance both *in vitro* and *in vivo*. Moreover, results illustrated that XR-2 possesses broad-spectrum anti-tumor activities *in vivo* with favourable safety.

**Conclusion:** MDM2-p53 inhibitor (XR-2) possesses potently prostate cancer progresses inhibition activity both *in vitro* and *in vivo*. XR-2 shows a synergistic effect with enzalutamide and overcomes enzalutamide resistance.

## Introduction

Globally, prostate cancer (PCa) is one of the most common malignancies and the fifth leading cause of cancer-related death in men. (Sung et al., 2021). Despite initial response to surgical, radiation and androgen ablation therapies, advanced localised PCa finally progresses to incurable metastatic castration-resistant PCa (CRPC). (Harris et al., 2009; Niu et al., 2010). Although second-generation anti-androgens (SGAs), which included abiraterone, enzalutamide, apalutamide and darolutamide, increased the overall survival time and decreased the prostate-specific antigen (PSA) level in patients with CRPC, (de Bono et al., 2011; Penson et al., 2016; Chi et al., 2019; Fizazi et al., 2020), 20–40% of these patients did not respond to abiraterone and enzalutamide. The remaining responders also inevitably developed acquired resistance, resulting in a limited survival improvement. (Chi et al., 2015; Wyatt et al., 2016). Many studies have confirmed that genomic aberrations, such as alterations of the androgen receptor (AR), DNA repair, p53 and Rb1, PI3K/AKT/mTOR pathway and Wnt/β-catenin pathway confer resistance to SGAs. (Watson et al., 2015; Ciccarese et al., 2017; Ku et al., 2017; Antonarakis et al., 2018; Isaacsson Velho et al., 2020). Based on molecular stratification, poly ADP-ribose polymerase (PARP) inhibitor prolongs survival time of advanced CRPC with certain DNA repair defects, leading to regulatory approvals in 2020. (Hussain et al., 2020). However, the BRCA1/2 mutation most sensitive to PARP inhibitor accounts for only a minority of CRPC cases with ATM mutations, comprising <10% of CRPC cases. (Neeb et al., 2021). Therefore, developing novel agents for men with fatal SGA-resistant CRPC is imperative.

The p53 is one of the most important tumor suppressors encoded by the *TP53* gene; p53 regulates some fundamental cellular processes under cellular stress, DNA damage or oncogene activation. (Wade et al., 2013; Levine, 2020). The transactivation of target genes by p53 induces cell cycle arrest, DNA repair and even apoptosis, and these downstream responses lead to the repair or culling of damaged and potentially tumorigenic cells. E3 ligase murine double minute-2 (MDM2) is a negative regulator of wild-type p53, which can promote p53 degradation through the proteasome pathway. The inactivation of p53 caused by p53 mutation or MDM2 overexpression in tumor cells is considered the main cause of tumor formation and progression. (Wade et al., 2013). Thus, the reactivation of p53 by blocking the MDM2–p53 interaction represents an attractive therapeutic remedy in cancers with wild-type or functional p53.

Inspiringly, a series of active small molecules have been developed by inhibiting MDM2–p53 interaction, with some currently being investigated in clinical trials regarding haematologic and solid malignancies. (Skalniak et al., 2019; Duffy et al., 2022). However, none of these inhibitors reached regulatory approval because gastrointestinal and bone marrow-related toxicities may restrict the clinical application of these drugs. (Konopleva et al., 2020). As a result, efforts to look for new-generation MDM2 inhibitors with stronger efficacy and acceptable toxicities are still worthwhile. Polyethylene glycol (PEG) modification is a well-known chemical modification strategy to increasing drug water solubility and reducing toxicities. Spirooxindole-containing compound is one of the most important MDM2 inhibitor types. By investigating different isomers of spirooxindole-containing compounds, Zhao et al. found that *cis-cis* isomers which contains *cis-cis* substitution pattern on the pyrrolidine ring showed high MDM2 binding affinity and complete long-lasting tumor regression in an animal model of human cancer, which shed light on MDM2 inhibitor development. (Zhao et al., 2013).

Previously, MDM2 inhibitor nutlin-3 and MI-219 were reported induced radiosensitization and enhanced androgen deprivation therapy (ADT) efficacy *in vitro* and *in vivo*. (Tovar et al., 2011; Feng et al., 2016). While, the influences of MDM2 inhibitor on SGAs therapeutic effect are still unanswered. Recent clinical studies have demonstrated that p53 inactivation was associated with poor response to SGAs. (Maughan et al., 2018; De Laere et al., 2019). In addition, *TP53* and *Rb1* play key roles in suppressing PCa lineage plasticity and anti-androgen resistance. (Mu et al., 2017; Nyquist et al., 2020). Therefore, we hypothesised that targeting p53 reactivation using MDM2 inhibitor could produce a synergistic effect with SGAs and potentially overcome SGAs resistance in PCa. In this study, we developed XR-2, which is a *cis-cis* isomer spirooxindole-based PEGylation MDM2 inhibitor, by specifically blocking MDM2–p53 interaction; XR-2 inhibits PCa proliferation in a p53-dependent manner. XR-2 displays a synergistic effect with enzalutamide and overcomes enzalutamide resistance both *in vitro* and *in vivo*. Furthermore, XR-2 possesses broad-spectrum anti-tumor activity *in vivo* in different cancer types with favourable safety.

## Methods

### Cell Lines and Reagents

The LNCaP (ATCC, CRL-1740), 22Rv1 (ATCC, CRL-2505), DU145 (ATCC, HTB-81), C4-2 (ATCC, CRL-3314), MCF7 (ATCC, HTB-22) and SJSA1 (ATCC, CRL-2098) cell lines were purchased from American Type Culture Collection (ATCC, VA, United States). The HepG2 (SCSP-510) and NCI-H460 (TCHu205) cell lines were purchased from the National Collection of Authenticated Cell Cultures of China. LNCaP, C4-2, 22Rv1, SJSA1 and NCI-H460 cell lines were cultured with Roswell Park Memorial Institute (RPMI)-1,640 supplemented with 10% foetal bovine serum (FBS, Gibco, Thermo Fisher Scientific, MA, United States). DU145 and HepG2 cell lines were cultured with DMEM supplemented with 10% FBS. MCF7 was cultured with Eagle’s minimum essential medium supplemented with 10% FBS. LNCaP-R cells were derived from LNCaP cells, as LNCaP cells were cultured with RPMI-1640 supplemented with 10% FBS and 5 μM enzalutamide for 2 months, and then these 5 μM enzalutamide-treated LNCaP cells were cultured with RPMI-1640 supplemented with 10% FBS and 10 μM enzalutamide for another 6 months.

XR-1, XR-2 and XR-3 (all produced in house) were dissolved in dimethyl sulfoxide (DMSO, Sigma-Aldrich, MO, United States) for *in vitro* experiments, and XR-2 was dissolved in Cremophor EL (Shanghai Aladdin Bio-Chem Technology Co., LTD., China), 95% ethanol (Shanghai Aladdin Bio-Chem Technology Co., LTD.) and saline solution (1.5:1.5:7) for *in vivo* experiments. Enzalutamide and RG7388 (idasanutlin) were purchased from Selleck (Houston, TX, United States).

### Western Blot Analysis

Logarithmic growth-phase cells were seeded at a density of ∼4 × 10^5^ cells per well in a 6-well plate and incubated for 48 h. Then, DMSO (Sigma-Aldrich) or test compounds were added to each well at the designated concentrations. After another 24 h of incubation, cells were collected and lysed with radio-immunoprecipitation assay lysis buffer supplemented with protease inhibitor and phosphatase inhibitor on ice for 30 min. Then, the protein lysis buffer was treated with 10% sodium dodecyl sulphate–polyacrylamide gel electrophoresis and transferred onto polyvinylidene difluoride membranes for Western blot analysis. The following antibodies were used for the detection of proteins: rabbit anti-MDM2 (1:1,000, Abways, Tracxn Technologies Limited, India), rabbit anti-p53 (1:1,000, Abways), mouse anti-PARP1 (1:1,000, Santa Cruz, United States), rabbit anti-cleaved PARP1 (1:1,000, Selleck), mouse anti-AR (441, 1:1,000, Santa Cruz, United States) and rabbit anti-PSA (1:1,000, Abways). Mouse anti-actin (1:5,000, Abcam) was used as a loading control. Proteins were visualised using anti-mouse, anti-rabbit or anti-rabbit HRP-conjugated secondary antibodies (1:5,000, Zhongshan Jinqiao Biotechnology Company, China) and ECL-Plus (Millipore, MA, United States). The resulting bands were analysed and quantified using ImageJ^®^ 1.49 g software (National Institutes of Health, MD, United States). Each experiment was repeated at least twice.

### Cell Viability Assay

PCa cells were seeded at a density of 1 ×10^4^ cells per well in a 96-well plate. LNCaP and 22Rv1 cells were cultured in a complete RPMI-1640 growth medium. DU145 cells were cultured in DMEM growth medium. After 24 h incubation, 1 µL of DMSO (Sigma-Aldrich) or 1 µl of the indicated concentrations of XR-2 were added to each well. After 72 h of incubation, 20 µl of MTT (Invitrogen, MA, United States) solution (5 mg/ml in phosphate-buffered saline (PBS)) was added per well and incubated for another 4 h. The MTT formazan formed by metabolically viable cells was dissolved in 100 µl of isopropanol. The absorbance was measured at 570 nm wavelength on a plate reader (EnSpire 2,300, PerkinElmer, MA, United States). Experiments were performed in triplicates. The value of the DMSO group was 100%.

### Colony Formation Assay

Logarithmic growth phase cells were seeded at a density of ∼1 ×10^4^ cells per well in 6-well plates (n = 3) and treated with the required drugs/compounds or vehicle for 12–14 days. The culture media were changed every 3 days. After removal of the culture media, cells were washed by PBS twice and colonies were fixed by methanol for 10 min. Thereafter, colonies were stained using 1% (w/v) crystal violet (Sigma, MO, United States) for 10 min. Each well was washed with distilled deionised water until the background was clean; after another 30 min of airing, colony pictures were generated. To quantify staining, the stained wells were washed with 1 ml of 10% acetic acid, and absorbance at 590 nm wavelength was detected on a plate reader (EnSpire 2,300, PerkinElmer).

### Quantitative Reverse-Transcriptase Polymerase Chain Reaction

All kinds of cells were seeded at a density of ∼4 × 10^5^ cells per well in a 6-well plate and incubated for 48 h. Then, cells were treated with vehicles (DMSO) or test compounds at designated concentrations. After another 24 h of incubation, total RNA was extracted by TRIzol (Invitrogen, MA, United States) according to the manufacturer’s instructions. Complementary cDNA synthesis was performed using a cDNA reverse transcription kit (AGbio, Inc.) and total mRNA templates. Relative mRNA levels of AR, PΜMA, p53 and p21 were quantified by qRT-PCR using a SYBR Green Premix Pro Taq HS qPCR Kit (AGbio) on the qPCR instrument (Bio-Rad, CA, United States). The mRNA expression levels were normalised to glyceraldehyde 3-phosphate dehydrogenase (GAPDH). All reactions were performed in triplicates. The gene-specific primers are listed in Supplementary Table S1.

### Gene Knocking Down Assay

The Small interfering RNAs (siRNAs) of *TP53* were designed and synthesized by Sangon Biotech (Shanghai, China). siRNA stocks (20 µM) and Lipofectamine™ RNAiMAX Transfection Reagent (ThermoFisher, 13778100) were diluted in opti-MEM, the mix was incubated for 20 min and then added to cells according to the instruction. After another 24 h, cells were treated with indicated compounds. The siRNA primers are listed in Supplementary Table S2.

### Flow Cytometry Analysis of Cell Cycle and Apoptosis

Logarithmic growth phase cells were seeded at a density of ∼4 ×10^5^ cells per well in a 6-well plate and incubated for 48 h. Then, DMSO (Sigma-Aldrich) or test compounds were added to each well at designated concentrations. For the cell cycle analysis, after compounds were treated for 24 h, cells were harvested and fixed with ice-cold 70% (v/v) ethanol for 24 h. Then, cells were centrifuged at 1,000 rpm for 5 min. Cell pellets were then washed once with phosphate-buffered saline (PBS) and stained with propidium iodide. A total of 30,000 events were acquired by flow cytometer (BDC6, BD Biosciences, United States), and proportions of cells in each phase of the cell cycle were calculated using FlowJo software (BD Biosciences). For apoptosis analysis, after compound treatment for 24 h, cells were harvested and washed once with cold PBS. Then, cells were incubated for 15 min at room temperature with Annexin V-FITC-PI in a binding buffer. Cells were then analysed on a flow cytometer (BDC6, BD Biosciences) using FlowJo software. Results were expressed as percentages of Annexin V+ cells. Experiments were performed in duplicate.

### Dual-Luciferase Reporter System Assay

As previously described, plasmid PSA-luc was a reporter gene plasmid in which the firefly luciferase expression is dependent on the PSA promoter; plasmid Renilla was a Ranilla luciferase reporter gene plasmid. These two plasmids were kindly provided by Dr Cen (Peking Union Medical College, China). LNCaP cells were seeded at a density of 6–7 × 10^4^ cells per well in 24-well plates. After incubation for 24 h, cells in each well were co-transfected with 100 ng of PSA-luc and 3 ng of Renilla plasmids using Lipofectamine 2000 reagent (Invitrogen) following the manufacturer’s protocol. After 24 h from the transfection, the medium was changed to phenol red-free RPMI-1640 supplemented with 10% charcoal-stripped FBS, containing 5 nM of DHT (1 µl) and 1 µl of test compounds at designated concentrations. After 24 h, the cells were lysed in 100 µl of passive lysis buffer per well, and 20 µl of the cell lysates were used for the detection of the luciferase activity using a dual-luciferase assay system (Promega, WI, United States) on a plate reader (Centro XS3 LB 960, Berthold Technologies, Germany). All experiments were run in triplicates.

### RNA Sequencing

LNCaP cells were treated by compounds at designated concentrations for 24 h. Total RNA was extracted from approximately 1 × 10^6^ cells using Invitrogen TRIzol^®^ RNA Isolation Reagent. Sequencing libraries were generated using VAHTS Universal V6 RNA-seq Library Prep Kit for Illumina^®^ (NR604-01/02, CA, United States), following the manufacturer’s recommendations. Index codes were added to attribute sequences to each sample. Then, the library was examined successively. The clustering of the index-coded samples was performed on a cBot cluster generation system using HiSeq PE Cluster Kit v4-cBot-HS (Illumina, CA, United States) according to the manufacturer’s instructions. After cluster generation, the libraries were sequenced on an Illumina platform and 150 bp paired-end reads were generated. The passed raw reads were aligned and assembled by STAR (https://github.com/alexdobin/STAR). Differentially expressed genes were analysed by HTseq-count (https://pypi.org/project/HTSeq) and edgeR (https://bioconductor.org/packages/edgeR). The KEGG analysis was performed by EnrichR (https://cran.rproject.org/). Heatmaps were generated by Hiplot website (https://hiplot.com.cn/). Original data is available in The Genome Sequence Archive for Human (https://ngdc.cncb.ac.cn/gsa-human/; Accession code: HRA001434).

### Senescence Assay

Cells were seeded at a density of ∼1 × 10^5^ cells per well in 6-well plates and incubated for 24 h. Then, DMSO or test compounds were added to each well at designated concentrations. After another 72 h of incubation, cell senescence was evaluated by visualising β-galactosidase activity using senescence β-galactosidase staining kit (C0602, Beyotime Technology, China) according to the manufacturer’s instructions. Three fields of each well were photographed for each of the three independent replicates for each treatment condition.

### *In vivo* Xenograft Studies

All animal experimental procedures were conducted in the animal facility of KeyGEN BioTECH (Nanjing, China). All experimental procedures involving the care and use of mice were approved by the KeyGEN BioTECH Institutional Animal Care and Use Committee. The mice were housed five per cage in an environmentally controlled SPF room (temperature 20–26°C; relative humidity 40–70%) on a 12-h light/dark cycle. The mice were fed commercial rodent chow (Beijing Keao Xieli Feed, Beijing, China) and received filter-purified water ad libitum. All studies utilised 4–6-week-old mice purchased from the Shanghai Laboratory Animal Center (Shanghai, China). 22Rv1 cells (2 × 10^6^ cells with Matrigel at a ratio of 1:1) were injected subcutaneously in the flank of the male SCID mice. SJSA-1 cells (2 × 10^6^ cells with Matrigel at a ratio of 1:1), NCI-H460 cells (2 × 10^6^ cells with Matrigel at a ratio of 1:1), or HepG2 cells (2 × 10^6^ cells with Matrigel at a ratio of 1:1) were injected subcutaneously in the flank of female BALB/c nude mice. When the average tumor volume reached 100–150 mm^3^, the mice were randomised and divided into the indicated groups (n = 8/group). XR-2 was administered once per day intraperitoneally. RG7388 and enzalutamide were administered once per day by oral gavage. Combination therapy studies were performed in a blinded manner. The tumor volume and mice bodyweight were monitored every other day, and the tumor volume was calculated according to the formula W^2^ × L/2 (mm^3^), wherein W was the short diameter and L was the long diameter. Data are expressed as mean ± SD. Whole blood was collected 24 h later after the last administration from the orbit. Blood cell analysis was performed using a haematology analyser (XS-800I, Sysmex Corporation, Japan).

### Immunohistochemical Staining

Antibodies to Ki67 (abcam, ab16667) and cleaved caspase 3 (CST, 9,661) were used for immunostaining on formalin-fixed paraffin-embedded xenograft tumor tissues. Generally, the rehydrated slides were microwave-heated for 20 min in citrate buffer (10-mM, pH 6.0) for antigen retrieval. Then incubated in 1% H_2_O_2_ for 10 min, after blocking with serum-free protein block, slides were incubated with the primary antibodies (Ki67, 1: 200; Cleaved caspase 3, 1: 100) for 2 h at room temperature, followed by incubation with HPR-conjugated secondary antibody for 1 h at room temperature. The immunoreaction products were visualized with 3, 3-diaminobenzidine (DAB)/H2O2 solution.

### In Silico Docking

The MDM2 protein crystal structure (PDB code: 5TRF) was downloaded from the PDB database (https://www.rcsb.org). Chain A of the MDM2 crystal structure remained and was modified by the “protonate 3D” module of Discovery Studio 3.5. The 2D chemical structure of XR-2 drawn by the Chemdraw 18.1 software was generated in 3D structure using the “prepare ligands” module by Discovery Studio 3.5. As described previously, (Li et al., 2019), the binding site was centred at Ile99 in MDM2 with a radius of 10 Å to cover the binding pocket of MDM2. Then, the prepared XR-2 has docked into the MDM2 chain A binding site by the ‘CDOCKER’ module of Discovery Studio 3.5 by default. After analysing the 10 binding poses of XR-2 to MDM2, we selected the highest-ranked pose for the MDM2 structure as the binding model of XR-2.

### Chemistry Synthesis

**2-(2-methoxyethoxy)ethyl-(2′S,3R,4′S,5′R)-5'-((4-carbamoyl-2-methoxyphenyl)carbamoyl**)**-6-chloro-4'-(3-chloro-2-fluorophenyl)-2′-neopentyl-2-oxospiro[indoline-3,3′-pyrrolidine]-1′-carboxylate (XR-1)**:Compound 2 was synthesized as previous explained. (Shu et al., 2013). Compound 2 (612 mg, 1.0 mmol) was dissolved in DMF (100 ml), then anhydrous KCO3 (4.0 mmol) was added and 1-chloroethyl (2- (2-methoxyethoxy) ethyl) carbonate (904 mg, 4.0 mmol) was added and stirred overnight at room temperature. After the reaction, water (20 ml) was added and extracted by ethyl acetate. The organic phase was separated, washed with brine, 1M hydrochloric acid and water successively, and then concentrated through the column to obtain the target product (644 mg, yield: 85%), which was a white solid. 1H NMR (CDCl3,400 MHz): 10.59 (s, 1H), 8.47 (d, J = 8.4 Hz, 1 H),7.80 (d, J = 1.6 Hz, 1 H), 7.55 (d, J = 1.3 Hz, 1 H), 7.48 (t, J = 6.8 Hz, 1 H), 7.28–7.36 (m, 2 H), 7.19–7.26 (m, 2 H), 7.04 (t, J = 8.0 Hz), 6.05–6.55 (bs, 1 H), 5.30–5.80 (bs, 1 H), 4.70 (t, J = 9.62 Hz, 1 H), 4.40–4.54 (m, 3 H), 3.94 (s, 3 H), 3.83 (t, J = 4.7 Hz, 2 H), 3.62–3.74 (m, 3 H), 3.60 (t, J = 4.6 Hz, 2 H), 1.24–1.35 (m, 1 H), 0.96 (s, 9 H); HRMS (ESI-TOF): m/z calculated for C37H41Cl2FN4NaO8+ [M + Na]+: 781.2183, found: 781.2190.

**2-(2-(2-methoxyethoxy)ethoxy**)**ethyl-(2′S,3R,4′S,5′R)-5'-((4-carbamoyl-2-methoxyphenyl)carbamoyl**)**-6-chloro-4'-(3-chloro-2-fluorophenyl)-2′-neopentyl-2-oxospiro[indoline-3,3′-pyrrolidine]-1′-carboxylate (XR-2):** Compound 2 (612 mg, 1.0 mmol) was dissolved in DMF (100 ml), then anhydrous KCO3 (4.0 mmol) was added with drops of 2- (2- (2-methoxyethoxy) ethane-1-ol (1.08 g, 4.0 mmol), and stirred overnight at room temperature. After the reaction, water (20 ml) was added and extracted by ethyl acetate. The organic phase was separated and washed with brine, 1M hydrochloric acid and water successively, and then concentrated through the column to obtain the target product (Yield: 80%), which was a white solid. 1H NMR (CDCl3,400 MHz): 10.6 (s, 1H), 8.45 (d, J = 8.0 Hz, 1H), 7.8 (s, 1 H), 7.55 (s, 1 H), 7.48 (t, J = 8.0 Hz, 1 H), 7.35 (t, J = 8.0 Hz, 2 H), 7.20–7.40 (m, 2 H), 7.04 (t, J = 8.0 Hz, 1 H), 6.1–6.6 (bs, 1 H), 5.4–5.8 (bs, 1 H), 4.69 (t, J = 10, 1 H), 4.40–4.55 (m, 3 H), 3.04 (s, 3 H), 3.83 (t, J = 4, 2 H), 3.69–3.77 (m, 4 H), 3.62–3.69 (m, 4 H), 3.50–3.58 (m, 2 H), 3.36 (s, 3 H), 3.18–3.30 (m, 1 H), 1.24–1.36 (m, 1 H), 0.95 (s, 9 H); 13C NMR (CDCl3,100 MHz): 174.41, 171.63, 168.82, 157.69, 155.21, 149.52, 148.46, 140.10, 134.91, 130.57, 130.01, 128.73, 127.35, 125.51, 124.81 (Jc-F = 4.3 Hz), 123.62, 123.36, 121.40 (d, J = 19 Hz), 119.95, 118.22, 116.15, 109.98, 72.01, 70.83, 70.81, 70.68, 68.61, 68.06, 66.60, 66.57, 65.51, 59.12, 55.76, 50.96, 42.79, 30.45, 29.89; HRMS (ESI-TOF): m/z calculated for C39H45Cl2FN4NaO9+ [M + Na]+: 825.2445, found: 825.2456.

**2,5,8,11,14-pentaoxahexadecan-16-yl-(2′S,3R,4′S,5′R)-5'-((4-carbamoyl-2-methoxyphenyl)carbamoyl**)**-6-chloro-4'-(3-chloro-2-fluorophenyl)-2′-neopentyl-2-oxospiro[indoline-3,3′-pyrrolidine]-1′-carboxylate (XR-3):** Compound 2 (306 mg, 0.5 mmol) was dissolved in DMF (50 ml), followed by the addition of anhydrous KCO3 (2.0 mmol) and the addition of 1-chloroethyl (2,5,8,11, 14-pentaxyhexadecane-16-yl) carbonate (720 mg, 2.0 mmol), stirred overnight at room temperature. After the reaction, water (20 ml) was added and extracted by ethyl acetate. The organic phase was separated, washed with brine, 1M hydrochloric acid and water successively, and then concentrated through the column to obtain the target product (Yield: 79%), which was a white solid. 1H NMR (CDCl3,400 MHz): 10.56 (s, 1 H), 8.41 (d, J = 8.4 Hz, 1 H), 7.79 (d, J = 1.7 Hz, 1 H), 7.54 (d, J = 1.3 Hz, 1 H), 7.49 (t, J = 6.8 Hz, 1 H), 7.20–7.40 (m, 4 H), 7.00–7.15 (m, 1 H), 6.30–6.60 (m, 1 H), 5.19 (bs, 1 H), 4.71 (t, J = 9.4 Hz, 1 H), 4.40–4.60 (m, 2 H), 4.25–2.34 (m, 1 H), 3.93 (s, 3 H), 3.83 (t, J = 4.6 Hz, 2 H), 3.78 (s, 1 H), 3.60–3.75 (m, 16 H), 3.50–3.58 (m, 2 H), 3.35–3.40 (m, 4 H), 1.20–1.37 (m, 1 H), 0.86–1.08 (m, 10 H); HRMS (ESI-TOF): m/z calculated for C43H53Cl2FN4NaO11 [M + Na]+: 913.2970, found: 913.2975.

### Data Analysis

Statistical analysis was performed using GraphPad Prism (version 5.01; GraphPad Software). Comparisons between groups were performed with a two-tailed Student’s t-test, except where specified, and differences were considered significant at *p* < 0.05.

## Results

### XR-2 as a Potent MDM2 Inhibitor

**FIGURE 1:**
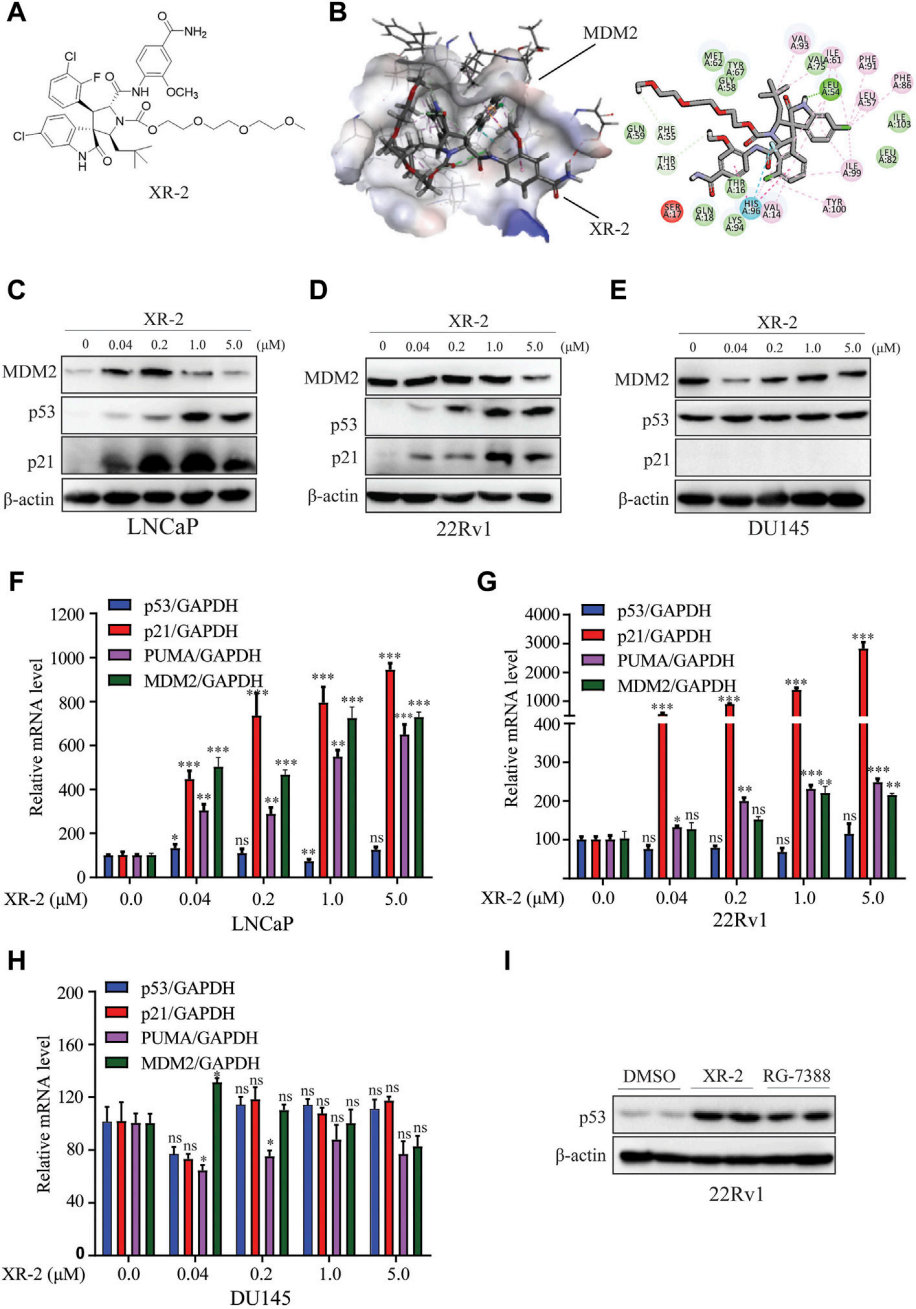
XR-2 effectively reactivates the p53 pathway. **(A)** Chemical structure of XR-2. **(B)** Predicted binding mode of XR-2 with MDM2. **(C)** Cells were treated with the indicated concentrations of XR-2 for 24 h. MDM2, p53, p21 and β-actin protein levels were measured by Western blot analysis in LNCaP, **(D)** 22Rv1 and **(E)** DU145 cells. **(F)** Cells were treated with the indicated concentrations of XR-2 for 24 h, and the mRNA levels of p53, *p21*, *PUMA* and *MDM2* were measured by qRT-PCR and normalised to GAPDH in LNCaP, **(G)** 22Rv1 and **(H)** DU145 cells. **(I)** 22Rv1 cells were treated with 1 μM XR-2 and 1 μM RG-7388 for 24 h; p53 and β-actin protein levels were measured by Western blot analysis. Experiments were performed in triplicates. All results are shown as mean ± SD. *p < 0.05, **p < 0.01, ***p < 0.001 vs. control group.

To develop a more potently selective MDM2 inhibitor with acceptable toxicity, we synthesised some of PEGylation spirooxindole derivatives (PSD) based on previous reported spiroindolinone pyrrolidinecarboxamide compound 2 (Supplementary Figure S1). (Shu et al., 2013). Then, we evaluated the primary tumor inhibition activity of some of these compounds in SJSA1-derived xenografts. The results indicated that the 2-weeks treatment with the *cis-cis* isomer of PSD XR-2 (Figure 1A) led to the highest suppression rate of tumor progression, which was higher than treatment with its *trans-cis* isomer and both isomers mixture. XR-2 was also proven to be more potent than its non-PEGylation initial analogue compound 2 (Supplementary Figure S2). Therefore, we chose XR-2 for further investigation.

To predict the binding mode of XR-2 to the MDM2 protein, molecular docking was performed based on the protein structure of MDM2 (PDB code: 5TRF) using Discovery Studio software. We found that XR-2 could suitably locate into the p53 binding site of MDM2 and form hydrogen bond interactions with Leu54 and His96 (Figure 1B). The homogeneous time-resolved fluorescence binding assay proved that XR-2 directly inhibited MDM2–p53 interaction (Supplementary Figure S3). Mechanistically, through competitive binding to the p53 pocket in MDM2, an MDM2 inhibitor could block MDM2–p53 interaction and subsequently led to p53 accumulation and transcriptional activation in wild-type p53 cells. Herein, we analysed the activity and specificity of XR-2 to activate p53 in PCa cells. In LNCaP cells with wild-type p53, Western blot analysis results indicated that XR-2 significantly induced p53 protein accumulation in a dose-dependent manner. In addition, we detected p21 protein upregulation, which further proved the activation of the p53 pathway (Figure 1C). In 22Rv1 cells, another wild-type p53 cells, XR-2 induced similar p53 activation (Figure 1D). However, in p53-mutated DU145 cells, XR-2 did not influence the levels of both p53 and p21 (Figure 1E). Moreover, quantitative reverse-transcriptase polymerase chain reaction (qRT-PCR) analysis revealed that XR-2 caused a dose-dependent upregulation of p53 downstream target genes *p21*, *PUMA* and *MDM2* but not p53 mRNA expression levels in both LNCaP and 22Rv1 cells (Figures 1F,G). Not surprisingly, XR-2 could not increase *p21*, *PUMA* and p53 mRNA levels in DU145 cells (Figure 1H). Notably, XR-2 demonstrated comparable p53 induction activity with the well-known MDM2 inhibitor RG7388 (Figure 1I). Taken together, our data provide clear evidence that XR-2 is a potent and specific MDM2 inhibitor.

### XR-2 Inhibits Cellular Proliferation and Induces Cell Cycle Arrest in Wild-Type p53 CRPC Cell Lines.

**FIGURE 2:**
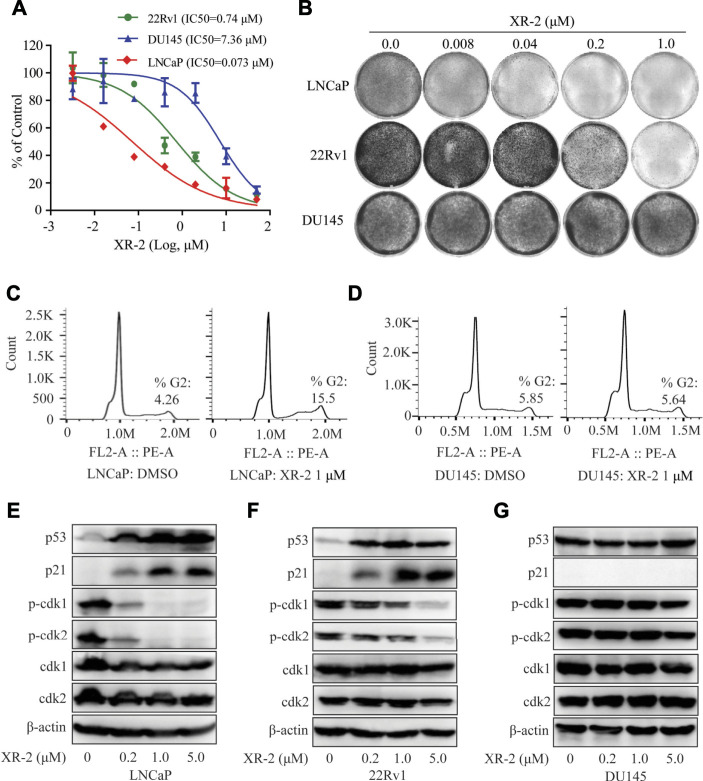
Influence of XR-2 on the cellular proliferation and cell cycle of CRPC cell lines. **(A)** CRPC cells, namely, LNCaP, 22Rv1 and DU145, were treated with different concentrations of XR-2 for 72 h, and cell proliferation was detected with the CCK8 assay. **(B)** Crystal violet staining of LNCaP, 22Rv1 and DU145 cells treated with different concentrations of XR-2 for 12–14 days **(C)** Flow cytometry assay was performed to detect cell cycle distribution of LNCaP cells treated with or without XR-2 for 24 h. **(D)** Flow cytometry assay was performed to detect cell cycle distribution of DU145 cells treated with or without XR-2 for 24 h. **(E)** Cells were treated with the indicated concentrations of XR-2 for 24 h; levels of p53, p21, p-CDK1, CDK1, p-CDK2, CDK2 and β-actin were measured by Western blot analysis in LNCaP, **(F)** 22Rv1 and **(G)** DU145 cells. Experiments were performed in triplicates. All results are shown as mean ± SD. *p < 0.05, **p < 0.01, ***p < 0.001 vs. control group.

The p53 key downstream target genes *p21* and *PUMA* could regulate cell cycle arrest, apoptosis and senescence in various cancer cells. Subsequently, we investigated the CRPC inhibition activity of XR-2; as shown in Figure 2A, XR-2 selectively inhibited the proliferation of wild-type p53 LNCaP and 22Rv1 cells in a dose-dependent manner with IC_50_ of 0.073 and 0.74 μM, respectively. Conversely, p53-mutated DU145 cells were quite less sensitive to XR-2. Furthermore, to estimate the effects of XR-2 on CRPC cell clonogenic activity, we exposed LNCaP, 22Rv1 and DU145 cells to different concentrations of XR-2 for about 2 weeks. As a result, XR-2 strongly decreased the number of LNCaP and 22Rv1 cell colonies in a dose-dependent manner compared with the untreated group, while specific treatment concentrations did not influence the number of DU145 cell colonies (Figure 2B).

Furthermore, flow cytometry analysis revealed that XR-2 therapy selectively increased the proportion of G2/M phase cells than the control group in LNCaP cells rather than in DU145 cells (Figures 2C,D). To further confirm the mechanisms of cell cycle arrest, we evaluated the influence of XR-2 on the protein levels of different cell cycle-related proteins in LNCaP, 22Rv1 and DU145 cells. XR-2 increased CDK suppressor p21 protein levels as mentioned above in LNCaP and 22Rv1 cells and thus dose-dependently reduced the cell cycle regulated protein levels of phosphorylation of CDK1 and CDK2 (Figures 2E,F). In DU145 cells, XR-2 could not upregulate p21 protein; thus, it cannot reduce the protein levels of activated phosphorylation CDK1 and CDK2 (Figure 2G). Collectively, these findings demonstrated that XR-2 effectively inhibited cell proliferation and induced cell cycle arrest of CRPC cells by activating the p53 pathway.

### XR-2 Promotes CRPC Cell Apoptosis and Senescence

**FIGURE 3:**
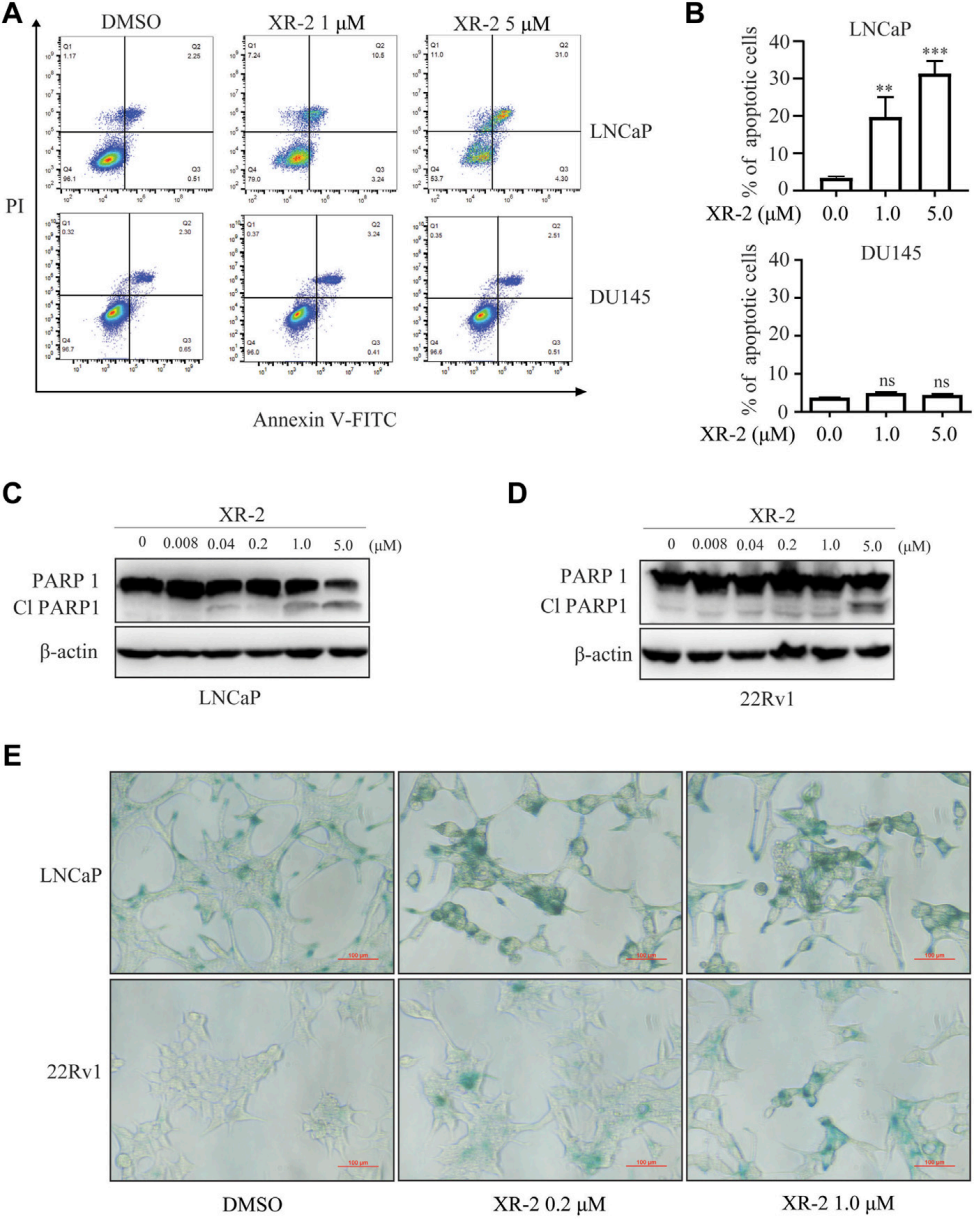
Influence of XR-2 on CRPC cell line apoptosis and senescence. **(A)** Flow cytometry assay to detect apoptosis levels of LNCaP and DU145 cells treated with different concentrations of XR-2 for 24 h. **(B)** Quantitative analysis of flow cytometry assay results. **(C)** Cells were treated with the indicated concentrations of XR-2 for 24 h, and PARP1 and β-actin protein levels were measured by Western blot analysis in LNCaP, and **(D)** 22Rv1 cells. **(E)** LNCaP and 22Rv1 cells were treated with the indicated concentrations of XR-2 for 72 h, and β-galactosidase staining was performed. Experiments were performed in triplicates. All results are shown as mean ± SD. *p < 0.05, **p < 0.01, ***p < 0.001 vs. control group.

Moreover, we examined whether XR-2 influences CRPC cell apoptosis by flow cytometry assay. As shown in Figures 3A,B, XR-2 significantly induced apoptosis of LNCaP cells rather than of DU145 cells. Western blot analysis further proved that XR-2 promoted the accumulation of apoptotic marker cleaved PARP protein in LNCaP and 22Rv1 cells (Figures 3C,D). The mRNA levels of apoptotic markers Bax and growth arrest and DNA damage-inducible 45 (GADD45A) were also upregulated in LNCaP and 22Rv1 cells rather than in DU145 cells (Supplementary Figure S4A,B). As we confirmed that XR-2 increased the expression of the senescence marker p21, we examined the mechanism of cell senescence by detecting senescence-associated β-galactosidase. We observed a significant increase in β-galactosidase staining in LNCaP and 22Rv1 cells treated with XR-2 for 72 h compared with vehicle treatment.

### XR-2 Inhibits CRPC Cell Viability in a p53-dependent Manner

**FIGURE 4:**
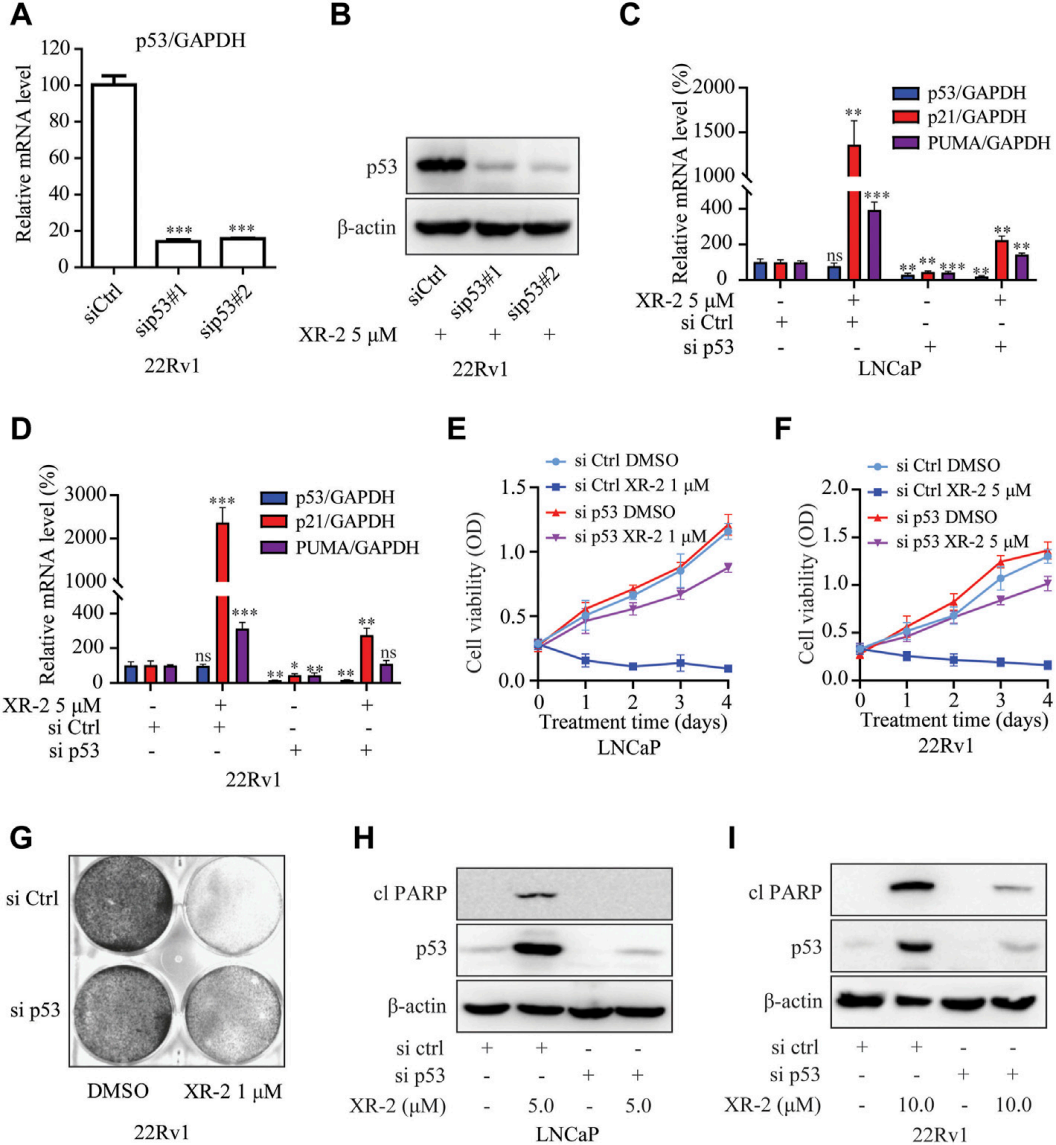
XR-2 inhibits CRPC cell viability through the p53 pathway. **(A)** 22Rv1 cells were treated with sip53 or siCtrl for 24 h, and the mRNA levels of p53 were measured by qRT-PCR and normalised to GAPDH. **(B)** 22Rv1 cells were treated with sip53 and XR-2 5 μM for 24 h, and p53 and β-actin protein levels were measured by Western blot analysis. **(C)** LNCaP cells were treated with sip53 and XR-2 1 μM for 24 h, and the mRNA levels of p53, p21 and *PUMA* were measured by qRT-PCR and normalised to GAPDH. **(D)** 22Rv1 cells were treated with sip53 and XR-2 5 μM for 24 h, and the mRNA levels of p53, *p21* and *PUMA* were measured by qRT-PCR and normalised to GAPDH. **(E)** LNCaP cells were treated with or without sip53 and XR-2 1 μM for different days, and cell proliferation was detected with the CCK8 assay. **(F)** 22Rv1 cells were treated with or without sip53 and XR-2 5 μM for different days, and cell proliferation was detected with the CCK8 assay. **(G)** Crystal violet staining of 22Rv1 cells treated with or without sip53 and XR-2 with 1 μM for 12–14 days **(H)** LNCaP cells were treated with or without sip53 and XR-2 with 5 μM for 24 h, and protein levels of cleaved PARP1, p53 and β-actin were measured by Western blot analysis. **(I)** 22Rv1 cells were treated with or without sip53 and XR-2 with 10 μM for 24 h, and protein levels of cleaved PARP1, p53 and β-actin were measured by Western blot analysis. Experiments were performed in triplicates. All results are shown as mean ± SD. *p < 0.05, **p < 0.01, ***p < 0.001 vs. control group.

To further confirm whether XR-2 inhibits CRPC cell viability through p53 activation, we performed *TP53* gene knockdown assay by siRNA. The results demonstrated that p53 knockdown reduced p53 mRNA expression levels in 22Rv1 cells (Figure 4A). Not surprisingly, XR-2 therapy was unable to promote p53 accumulation in p53 knockdown cells (Figure 4B). Efficient knockdown of p53 in LNCaP and 22Rv1 cells also potently attenuated XR-2 induced upregulation of *p21* and *PUMA* mRNA expression levels (Figures 4C,D). Furthermore, p53 knockdown forcefully reduced the cell proliferation inhibition potency of XR-2 in LNCaP and 22Rv1 cells (Figures 4E,F). We also found that XR-2-induced colony formation inhibition activity in 22Rv1 cells was blocked by p53 knockdown (Figure 4G). Finally, we found that p53 knockdown diminished XR-2-induced cell apoptosis indicated by the expression levels of the cleaved PARP protein in LNCaP and 22Rv1 cells (Figures 4H,I). These data firmly established that XR-2 inhibits CRPC cell viability through the p53 pathway.

### XR-2 Inhibits the AR Pathway and Shows Synergistic Effects With Enzalutamide

**FIGURE 5:**
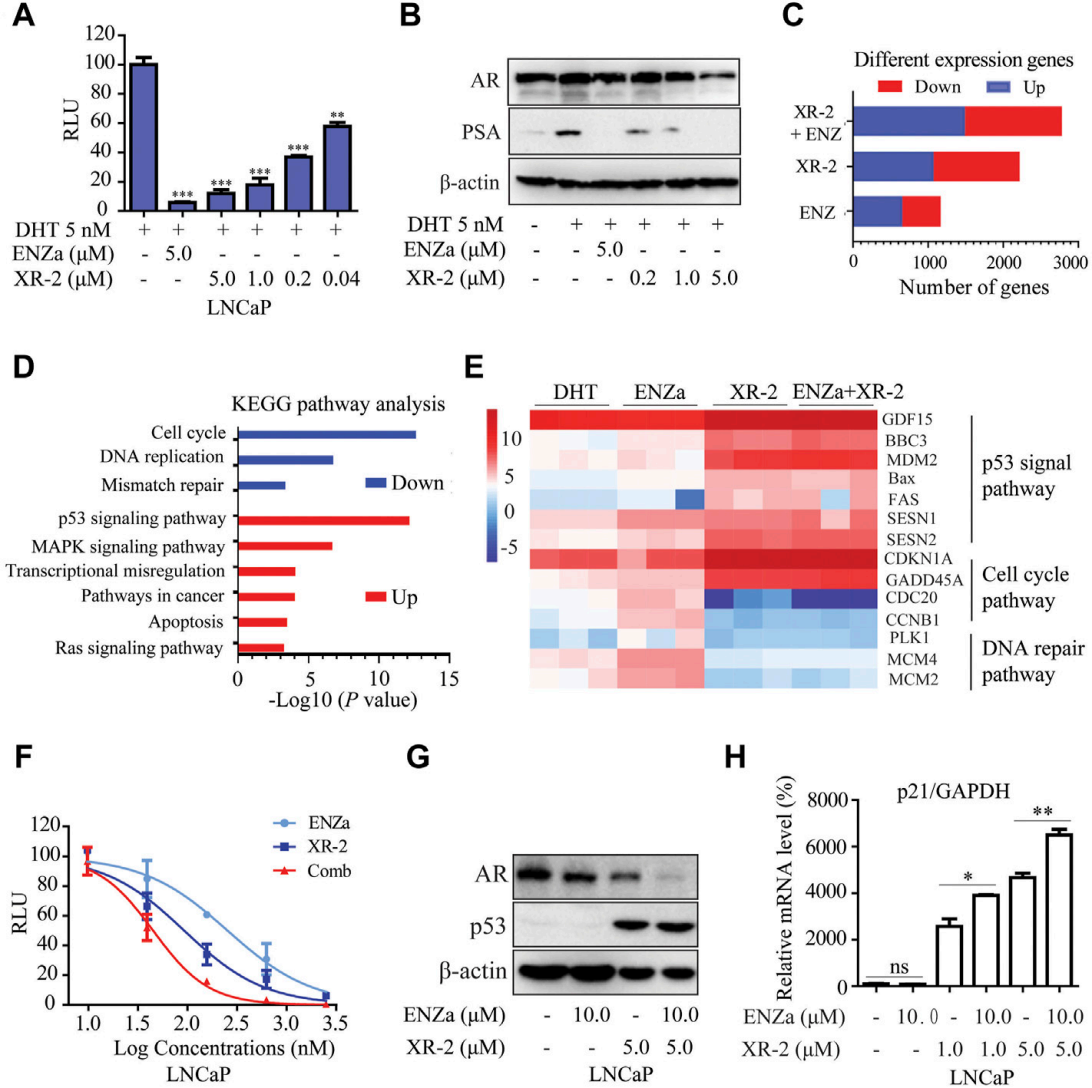
XR-2 blocks the AR pathway and demonstrates synergistic effects with enzalutamide. **(A)** A dual-luciferase reporter assay was performed to measure PSA-luc reporter luciferase activities stimulated by 5 nM DHT and treated with different concentrations of XR-2 for 24 h in LNCaP cells co-transfected with the Renilla and PSA promoter expression vector plasmids. **(B)** LNCaP cells were treated with the indicated concentrations of XR-2 in the presence of 5 nM DHT for 24 h, cells were then lysed, and PSA and AR protein levels were measured by Western blot analysis. **(C)** LNCaP cells were treated with XR-2 (1 μM), enzalutamide (5 μM), alone or in combination for 24 h in triplicate experiments. Cells were collected for RNA-seq analysis after treatment. The number of differentially expressed genes after each treatment was shown. **(D)** The Kyoto Encyclopedia of Genes and Genomes pathway analysis of the most significantly enriched biological processes upregulated or downregulated in response to combined treatments compared with control groups. **(E)** Representative differentially expressed genes in response to XR-2, enzalutamide and combination treatments. **(F)** A dual-luciferase reporter assay was performed to measure PSA-luc reporter luciferase activities stimulated by 5 nM DHT and treated with different concentrations of XR-2, enzalutamide or both agents combined for 24 h in LNCaP cells co-transfected with the Renilla and PSA promoter expression vector plasmids. **(G)** LNCaP cells were treated with XR-2, enzalutamide or both agents combined for 24 h, cells were then lysed and p53 and AR protein levels were measured by Western blot analysis. **(H)** LNCaP cells were treated with XR-2, enzalutamide or both agents combined for 24 h, and the mRNA levels of *p21* were measured by qRT-PCR and normalised to GAPDH. Experiments were performed in triplicates. All results are shown as mean ± SD. *p < 0.05, **p < 0.01, ***p < 0.001 vs. control group.

As p53 overexpression was reported to diminish the androgen response, (Cronauer et al., 2004), we investigated whether p53 inducer XR-2 could block the AR pathway in PCa cells. The results of dual-luciferase reporter assay in LNCaP cells indicated that XR-2 inhibited dihydrotestosterone (DHT)-induced transcriptional activities of endogenous AR in a dose-dependent manner (Figure 5A). Moreover, Western blot analysis revealed that XR-2 downregulated DHT-activated PSA protein expression levels in a dose-dependent manner; surprisingly, XR-2 could also downregulate AR protein levels (Figure 5B).

RNA sequencing assay was performed to evaluate the gene expression status of LNCaP cells under XR-2 and enzalutamide treatment. Compared with single agents, XR-2 plus enzalutamide in LNCaP cells resulted in more differentially expressed genes, implicating that combined treatment had more remarkable effects (Figure 5C). The Venn analysis indicated that the largest number of differentially upregulated and downregulated genes appeared to be induced by XR-2 plus enzalutamide compared with either single-agent treatment. Interestingly, the proportion of overlapped genes between XR-2 monotherapy and combination therapy appeared higher than that between enzalutamide monotherapy and combination therapy (Supplementary Figure S5A,B). The Kyoto Encyclopedia of Genes and Genomes (KEGG) analysis demonstrated that XR-2 plus enzalutamide upregulated the expression of genes encoding constituents of the P53 signalling pathway and downregulated those involved in DNA replication, mismatch repair and cell cycle progression (Figure 5D). As shown in Figure 5E, the main p53-target genes were upregulated in XR-2 monotherapy and combination therapy, such as growth differentiation factor 15 (GDF15), which is a surrogate for p53 activation, and apoptogenic genes *BBC3*, *PUMA*, *MDM2*, *Bax* and *FAS*. XR-2 monotherapy and combination therapy also upregulated the expressions of negative regulators of cell cycle progression genes, including *cyclin-dependent kinase inhibitor 1A* and *GADD45*. In addition, the combination therapy enhanced AR pathway inhibition compared with the control and enzalutamide groups, which were characterised by lower expression levels of key AR-target genes including *FKBP5*, *PMEPA1*, *KLK3*, *NKX3-1* and *KLK2* (Supplementary Figure S5C). Altogether, these findings suggest that XR-2 plus enzalutamide induced enhanced p53 pathway reactivation and AR pathway inhibition.

To further confirm the synergistic effects between XR-2 and enzalutamide, we examined the influences of XR-2 plus enzalutamide on AR and p53 pathways. We found that XR-2 plus enzalutamide demonstrated enhanced AR transcriptional inhibition activity than either monotherapy in different concentrations (Figure 5F). Similarly, as demonstrated in Figure 5G, the XR-2 plus enzalutamide reduced AR protein levels more efficiently than monotherapy in both LNCaP, 22Rv1 and C4-2 cells (Supplementary Figure S5D,F). By contrast, by detecting the *p21* mRNA levels in LNCaP, 22Rv1 and C4-2 cells, we found that enzalutamide could enhance XR-2-induced p53 pathway activation (Supplementary Figure S5G,H,E). Together, these findings indicate that XR-2 could inhibit the AR pathway, and XR-2 plus enzalutamide synergistically inhibits AR and p53 pathways.

### XR-2 Overcomes Enzalutamide Resistance *in vitro*

**FIGURE 6:**
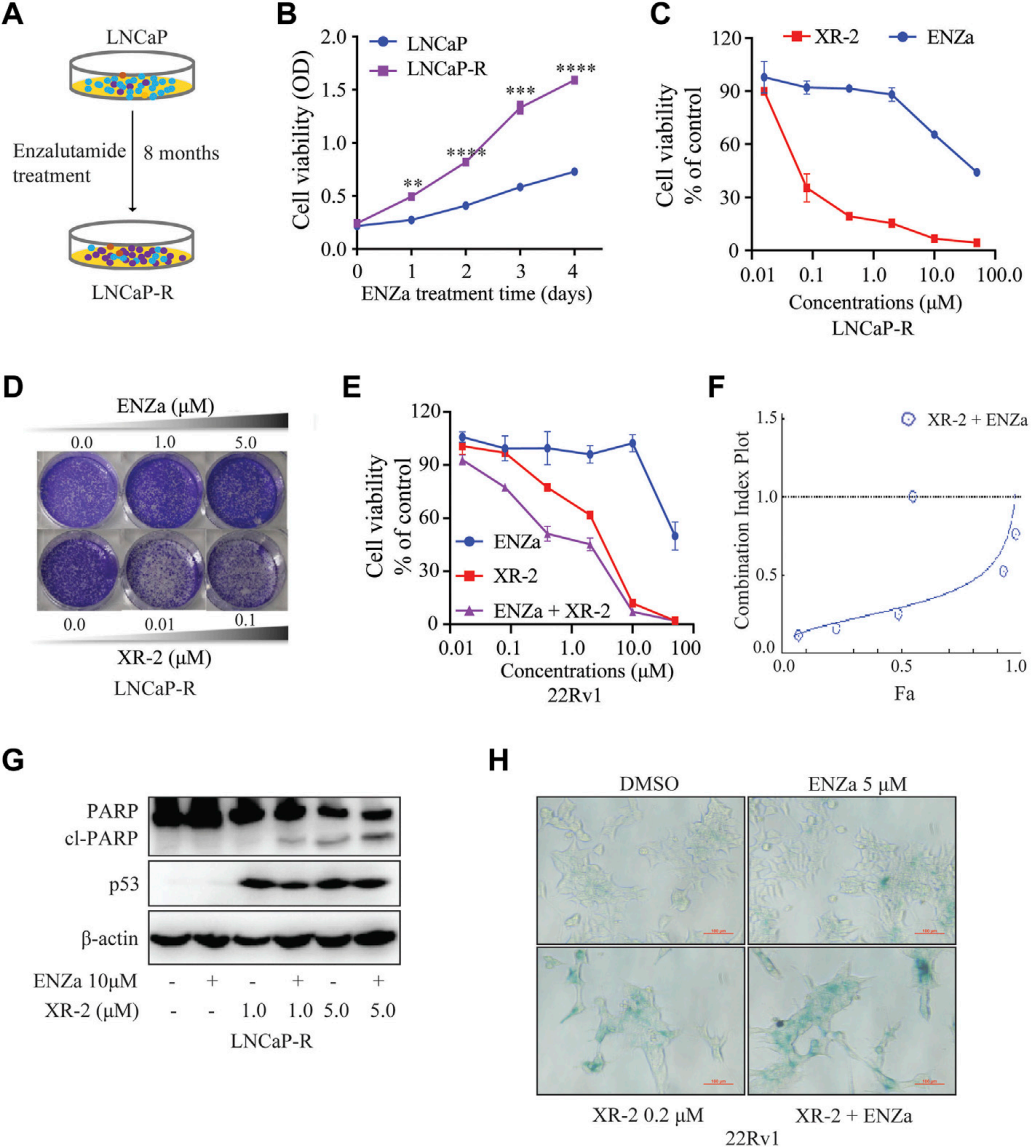
XR-2 inhibits the viability of enzalutamide-resistant CRPC cells. **(A)** Sketch map about the construction of LNCaP-R cells. **(B)** LNCaP and LNCaP-R cells were treated with enzalutamide 5 μM for days, and cell proliferation was examined with the CCK8 assay. **(C)** LNCaP-R cells were treated with different concentrations of XR-2 and enzalutamide for 72 h, and cell proliferation was assessed with the CCK8 assay. **(D)** Crystal violet staining of LNCaP-R cells treated with different concentrations of XR-2 and enzalutamide for 12–14 days **(E)** 22Rv1 cells were treated with different concentrations of XR-2, enzalutamide or both agents combined for 72 h, and cell proliferation was evaluated with the CCK8 assay. **(F)** The combination index of XR-2 and enzalutamide was calculated by CompuSyn software. **(G)** LNCaP-R cells were treated with XR-2, enzalutamide or both agents combined for 24 h, and PARP1, p53 and β-actin protein levels were measured by Western blot analysis. **(H)** 22Rv1 cells were treated with the indicated concentrations of XR-2, enzalutamide or both agents combined for 72 h, and β-galactosidase staining was performed. Experiments were performed in triplicates. All results are shown as mean ± SD. *p < 0.05, **p < 0.01, ***p < 0.001 vs. control group.

To evaluate whether XR-2-induced p53 reactivation and AR inhibition could inhibit enzalutamide-resistant CRPC cell viability, we constructed enzalutamide-resistant LNCaP (LNCaP-R) cells through long-term enzalutamide treatment (Figure 6A). Compared with LNCaP cells, LNCaP-R cells demonstrated a more robust cell proliferation activity with 5 μM enzalutamide treatment (Figure 6B). Nevertheless, XR-2 potently blocked LNCaP-R cell proliferation and colony formation activity in a dose-dependent manner (Figures 6C,D). In enzalutamide-resistant 22Rv1 cells, XR-2 similarly suppressed the proliferation of 22Rv1 cells and demonstrated synergistic effects with enzalutamide (Figures 6E,F). Western blot analysis revealed that XR-2 also induced apoptosis marker protein cleaved PARP1 expression and demonstrated synergistic effects with enzalutamide in LNCaP-R cells (Figure 6G). Subsequently, we found that the proportion of β-galactosidase-positive cells were significantly increased in both XR-2 monotherapy and combination therapy compared with that in the control or enzalutamide group. These results proved that XR-2 could overcome enzalutamide resistance *in vitro*.

### XR-2 Inhibits Enzalutamide-resistant CRPC Xenograft Progress

**FIGURE 7:**
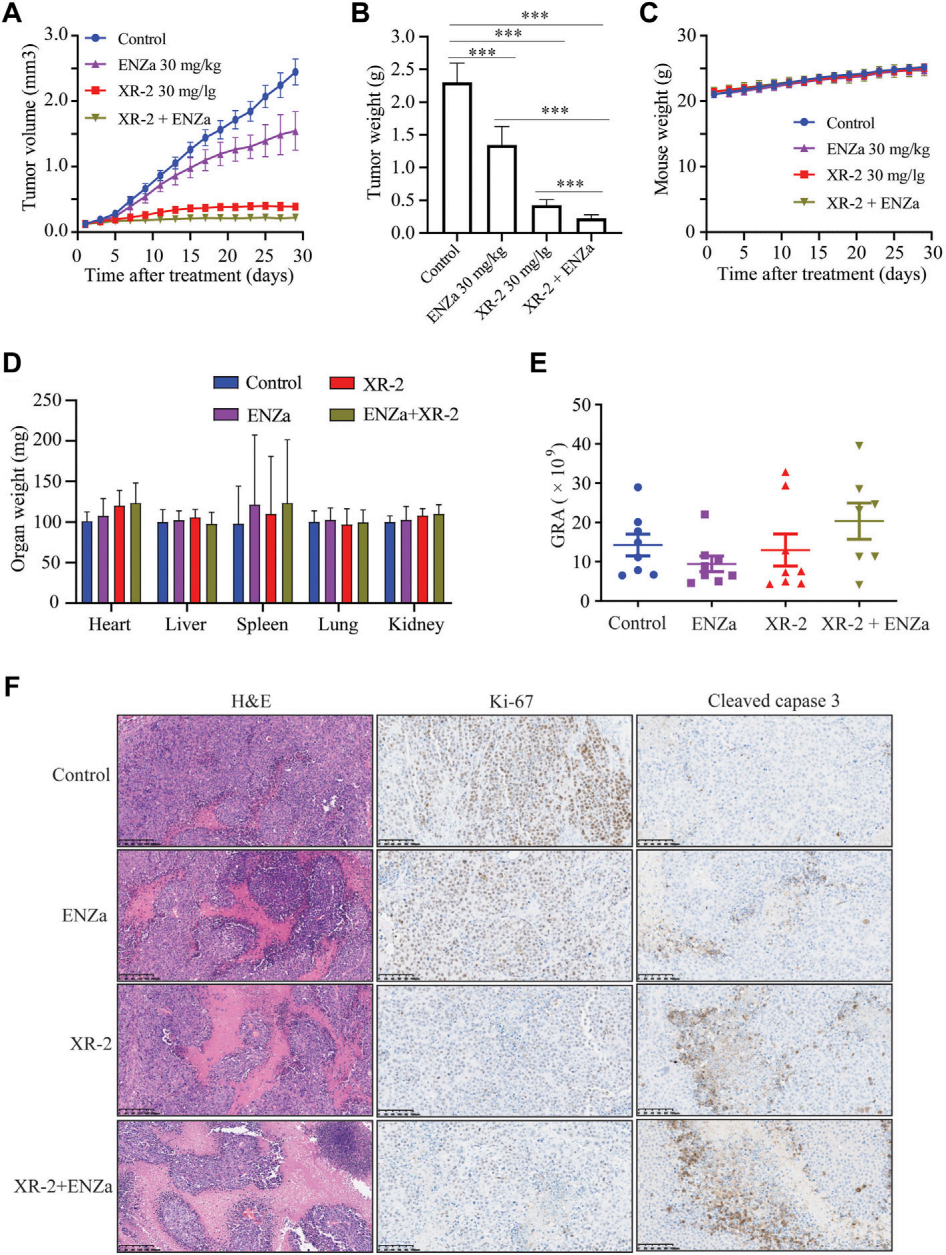
XR-2 suppresses 22Rv1 cell-derived xenograft progress *in vivo*. **(A)** Xenografts arising from 22Rv1 cells were treated with blank control, 30 mg/kg of XR-2, 30 mg/kg of enzalutamide and 30 mg/kg of XR-2 plus 30 mg/kg enzalutamide once a day for 4 weeks, and tumor growth was monitored every other day. **(B)** Tumor weight at the last observation day. **(C)** Mice were weighed by electronic scale every other day. **(D)** Critical organs of the mice were weighed at the last observation day. **(E)** Blood neutrophil counts of the mice at the last observation day. **(F)** Immunohistochemical analysis of Ki67 and cleaved caspase 3 levels in harvested tumor s. All results are shown as mean ± SD. *p < 0.05, **p < 0.01, ***p < 0.001 vs. control group.

As previous study proved that XR-2 could activate the p53 pathway and inhibit PCa cell viability *in vitro*. Then, we investigated the tumor inhibition efficacy of XR-2, enzalutamide and XR-2 plus enzalutamide *in vivo* by using 22Rv1 xenografts in male SCID mice. In this study, 5 ×10^6^ 22Rv1 cells were injected into the left flank of each male mouse, and the tumor volume was allowed to increase to approximately 100 mm^3^. Then, the tumor-bearing mice received intraperitoneal injection of the vehicle control, 30 mg/kg of XR-2, 30 mg/kg of enzalutamide and 30 mg/kg of XR-2 plus 30 mg/kg of enzalutamide once a day for 4 weeks. The results indicated that enzalutamide could only inhibit 22Rv1 tumor growth weakly, mainly due to ARV7 overexpression, while both XR-2 monotherapy and combination therapy suppressed 22Rv1 tumor progression and decreased tumor weight significantly (Supplementary Figure S6A, S7A,B). Interestingly, the combination therapy revealed a more effective tumor inhibition activity than XR-2 or enzalutamide monotherapy. We also evaluated whether XR-2 treatment activated p53 protein levels *in vivo*; as shown in Supplementary Figure S6B, the tumor p53 protein levels were remarkably accumulated in both XR-2 monotherapy and combination therapy groups. Moreover, XR-2 was tolerable to the SCID mice, as the mice bodyweight of the XR-2 monotherapy and combination therapy groups nearly had no change compared with the vehicle group (Figure 7C). Further analysis revealed that both XR-2 monotherapy and combination therapy did not influence the weight of the critical organs and blood neutrophil counts of mice, which reminded the favourable *in vivo* safety of XR-2 (Figures 7D,E). Moreover, immunohistochemistry assay results proved that XR-2 reduced Ki-67 protein levels and induced cleaved caspase3 protein accumulation compared with the vehicle group. Compared with monotherapy, the combination therapy enhanced these changes in protein levels (Figure 7F). Taken together, our data indicated that XR-2 could inhibit the growth of CRPC both *in vitro* and *in vivo*.

## Discussion

Drug resistance such as AR pathway alterations, oncogene activation and lineage plasticity have been widely reported to restrict the clinical benefits of SGAs. (Watson et al., 2015; Ge et al., 2020; Isaacsson Velho et al., 2020). Studies have demonstrated that the inactivation of the tumor suppressor p53 was associated with poor response to SGAs and lineage plasticity in PCa. (De Laere et al., 2019; Nyquist et al., 2020). In this work, we identified a compound XR-2 that effectively inhibits MDM2–p53 interaction and selectively reactivates the p53 pathway. XR-2 could suppress the proliferation activity of CRPC cells in a p53-dependent manner. Besides, XR-2 induces cell cycle arrest and apoptosis in wild-type p53 CRPC cell lines. Further study proves that XR-2 could also block the AR pathway and shows a synergistic effect with enzalutamide through p53 pathway reactivation and AR pathway inhibition. Moreover, XR-2 could overcome enzalutamide resistance both *in vitro* and *in vivo*. These results tend to support the potential of XR-2 monotherapy and XR-2 plus enzalutamide in the treatment of SGA-resistant CRPC.

MDM2 overexpression and p53 mutation in PCa is associated with worse clinical outcomes. MDM2 overexpression was a prognostic of the development of metastatic disease as well as overall mortality. (Khor et al., 2009; Venkatesan et al., 2018). *TP53* inactivation was established as a biomarker to predict abiraterone or enzalutamide resistance in metastatic CRPC. (Maughan et al., 2018; De Laere et al., 2019). Therefore, the above observations suggest that MDM2 inhibition in PCa may have a dual suppressive effect by blocking MDM2 function and activating p53 functions. Thus, the inhibition of MDM2 represents an attractive strategy for the treatment of PCa with wild type p53, especially in combination with the current standard of care therapies. Efforts had been made to evaluate the effects of MDM2–p53 inhibitors in PCa treatment. The first-generation MDM2 inhibitor Nutlin-3 could inhibit androgen receptor-driven c-FLIP expression, resulting in apoptosis of PCa cells and enhancing the curative effect of chemotherapy. Another MDM2 inhibitor MI-219 showed the sensitisation of PCa cells to radiotherapy and androgen deprivation therapy. (Chappell et al., 2012; Feng et al., 2016; Logan et al., 2016). However, whether MDM2-p53 inhibitor could combine SGAs to treat CRPC is still unanswered. Previous work has proved that p53 could inhibit AR expression levels through combining with p53 DNA-binding site of *AR* gene. (Alimirah et al., 2007; Chopra et al., 2018). Our work indicates that XR-2 is able to induce p53 accumulation in wild-type p53 PCa cells, as a result, accumulated p53 downregulates the AR protein expression levels. Moreover, MDM2 E3 ligase activity is reported to play vital role in proteasome-mediated AR ubiquitylation and degradation. (Lin et al., 2002). Many researches have demonstrated that MDM2-p53 inhibitors could upregulate MDM2 expression levels in wild-type p53 cancer cells, our work also finds XR-2 upregulates MDM2 levels in PCa cells, therefore, promoting MDM2-regulated AR and ARV7 degradation through proteasome pathway. Theoretically, these mechanisms may explain the combination of MDM2-p53 inhibitor XR-2 and AR antagonist enzalutamide induces enhanced AR pathway inhibition. All in all, the AR downregulating effects of XR-2 combine with the AR antagonizing effects of enzalutamide contribute to the role of the combination in overcoming AR pathway alterations such as AR mutation, AR overexpression and AR splice variants induced by SGA drug resistance.

Despite clinical trials of some MDM2–p53 inhibitors in haematologic and solid malignancies, none of these inhibitors reached regulatory approval and mainly imputed gastrointestinal and bone marrow-related toxicities. (Konopleva et al., 2020). In this study, we develop a novel *cis-cis* isomer of spirooxindole-based PEG modification MDM2 inhibitor XR-2, which has favourable water solubility, so that parenteral administration partially reduced irritation to the gastrointestinal tract. Blood cell analysis also proves that XR-2 does not affect the neutrophil count. MDM2–p53 inhibitors have broad-spectrum anti-tumor activity in many other studies. (Yi et al., 2018; Fang et al., 2020). XR-2 also demonstrated anti-tumor activity *in vivo* in various tumor types. In NCI-H460, which is a p53 wild-type lung cancer cell line model, XR-2 suppressed NCI-H460 tumor progression effectively, and XR-2 did not delay the weight gain of mice that received the therapeutic dose. Similarly, XR-2 could significantly inhibit the progression of liver cancer cell line HepG2-derived tumor *in vivo* with acceptable safety. More importantly, in a p53 wild-type osteosarcoma cell line SJSA1-derived xenograft model, XR-2 shows stronger tumor inhibition activity than RG-7388, which is under clinical investigation. (Ding et al., 2013). XR-2 nearly lost its influence on mouse weight even under treatment with 100 mg/kg dose, which converts to a human equivalent dose of approximately 500 mg for a 60-kg patient. These data illustrate the safety and effectiveness of XR-2 in cancer treatment.

## Conclusion

MDM2 inhibitor (XR-2) possesses potently prostate cancer progresses inhibition activity both *in vitro* and *in vivo*. XR-2 shows a synergistic effect with enzalutamide and overcomes enzalutamide resistance. This is the first report on MDM2-p53 inhibitor overcoming SGAs resistance which provide convincing clues for further clinical trial of the combination therapy of SGAs with MDM2 inhibitor in prostate cancer.

## Abbreviationsabbreviation

MDM2, modification murine double minute-2; qRT-PCR, quantitative reverse-transcriptase polymerase chain reaction; GAPDH, glyceraldehyde 3-phosphate dehydrogenase. DHT, dihydrotestosterone; CRPC, castration-resistant prostate cancer Contributions.

## Data Availability

The datasets presented in this study can be found in online repositories. The names of the repository/repositories and accession number(s) can be found in the article/Supplementary Material.

## Supplementary Material

The Supplementary Material for this article can be found online at: https://www.frontiersin.org/articles/10.3389/fphar.2022.871259/full#supplementary-material

Supplementary Figure S1 The procedure for the synthesis of compound XR-2 and its analogues.

Supplementary Figure S2 Primary evaluation of pegylation spirooxindole derivatives (PSD) tumor inhibition activity *in vivo*. **(A)** The stereochemical structure of *cis-cis* isomer XR-2 and its *trans-cis* isomer XR-2-2. **(B)** Xenografts arising from SJSA1 cells were treated with blank control, 30 mg/kg compound 2, 30 mg/kg XR-2 (racemic), XR-2 (laevo) and XR-2 (dextro) once a day for 14 days, tumour growth was monitored every other day. **(C)** Mice image at the last monitor day. **(D)** Tumour image at the last monitor day.

Supplementary Figure S3 The effects of XR-2 on MDM2-p53 binding activity were detected by HTRF assay. Experiments were in triplicates. All results are shown as mean ± s.d.

Supplementary Figure S4 The influence of XR-2 on CRPC cell lines apoptosis. **(A, B)** LNCaP, 22Rv1 and DU145 cells were treated with DMSO and XR-2 5 μM for 24 h, the mRNA levels of *Bax* and *GADD45A* were measured by quantitative-PCR and normalized to GAPDH. Experiments were in triplicates. All results are shown as mean ± s.d. **P* < 0.05, ***P* < 0.01, ****P* < 0.001 vs. control group.

Supplementary Figure S5 XR-2 blocks AR pathway and shows synergistic effects with enzalutamide. **(A)** Venn Diagram about up-regulated numbers of differentially expressed genes after each treatment. **(B)** Venn Diagram about down-regulated numbers of differentially expressed genes after each treatment. **(C)** Representative AR pathway genes in response to enzalutamide, and combination treatment. **(D)** 22Rv1 cells were treated with XR-2, enzalutamide or both agents combined for 24 h, then cells were lysed, p53 and AR protein levels were measured by western blot analysis. **(E)** 22Rv1 cells were treated with XR-2, enzalutamide or both agents combined for 24 h, the mRNA levels of *p21* were measured by quantitative-PCR and normalized to GAPDH. **(F)** C4-2 cells were treated with XR-2, enzalutamide or both agents combined for 24 h, then cells were lysed, p53 and AR protein levels were measured by western blot analysis. **(G)** C4-2 cells were treated with XR-2, enzalutamide or both agents combined for 24 h, the mRNA levels of *p21* were measured by quantitative-PCR and normalized to GAPDH. Experiments were in triplicates. All results are shown as mean ± s.d. **P* < 0.05, ***P* < 0.01, ****P* < 0.001 vs. control group.

Supplementary Figure S6 XR-2 suppresses 22Rv1 cells derived xenografts progress *in vivo*. **(A)** Xenografts arising from 22Rv1 cells were treated with blank control, 30 mg/kg XR-2, 30 mg/kg enzalutamide and 30 mg/kg XR-2 combined with 30 mg/kg enzalutamide once a day for 4 weeks, tumor image at the last monitor day was photographed. **(B)** 22Rv1 cells derived tumors were lysed by RIPA buffer, p53 and GAPDH protein levels were measured by western blot analysis.

Supplementary Figure S7 XR-2 suppresses wide types cancer cells derived xenografts progress *in vivo*. **(A)** Xenografts arising from NCI-H460 cells were treated with blank control and 30 mg/kg XR-2 once a day for 31 days, tumor growth was monitored every other day. **(B)** Mice image at the last monitor day. **(C)** Mice weight was weighed by electronic scale every other day. **(D)** Xenografts arising from HepG2 cells were treated with blank control and 30 mg/kg XR-2 once a day for 28 days, tumor growth was monitored every other day. **(E)** Mice image at the last monitor day. **(F)** Mice weight was weighed by electronic scale every other day. **(G)** Xenografts arising from SJSA1 cells were treated with blank control, 50 mg/kg XR-2, 100 mg/kg XR-2 and 50 mg/kg RG-7388 once a day for 14 days, tumor growth was monitored every other day. **(H)** Mice image at the last monitor day. **(I)** Mice weight was weighed by electronic scale every other day. All results are shown as mean ± s.d. **P* < 0.05.

## Abbreviations

MDM2, modification murine double minute-2; qRT-PCR, quantitative reverse-transcriptase polymerase chain reaction; GAPDH, glyceraldehyde 3-phosphate dehydrogenase. DHT, dihydrotestosterone; CRPC, castration-resistant prostate cancer Contributions.

## References

[B1] Alimirah F. Panchanathan R. Chen J. Zhang X. Ho S. M. Choubey D. (2007). Expression of Androgen Receptor Is Negatively Regulated by P53. Neoplasia 9 (12), 1152–1159. 10.1593/neo.07769 18084622 PMC2134911

[B2] Antonarakis E. S. Lu C. Luber B. Liang C. Wang H. Chen Y. (2018). Germline DNA-Repair Gene Mutations and Outcomes in Men with Metastatic Castration-Resistant Prostate Cancer Receiving First-Line Abiraterone and Enzalutamide. Eur. Urol. 74 (2), 218–225. 10.1016/j.eururo.2018.01.035 29439820 PMC6045965

[B3] Chappell W. H. Lehmann B. D. Terrian D. M. Abrams S. L. Steelman L. S. McCubrey J. A. (2012). p53 Expression Controls Prostate Cancer Sensitivity to Chemotherapy and the MDM2 Inhibitor Nutlin-3. Cell Cycle 11 (24), 4579–4588. 10.4161/cc.22852 23187804 PMC3562303

[B4] Chi K. Hotte S. J. Joshua A. M. North S. Wyatt A. W. Collins L. L. (2015). Treatment of mCRPC in the AR-axis-targeted Therapy-Resistant State. Ann. Oncol. 26 (10), 2044–2056. 10.1093/annonc/mdv267 26101426

[B5] Chi K. N. Agarwal N. Bjartell A. Chung B. H. Pereira de Santana Gomes A. J. Given R. (2019). Apalutamide for Metastatic, Castration-Sensitive Prostate Cancer. N. Engl. J. Med. 381 (1), 13–24. 10.1056/NEJMoa1903307 31150574

[B6] Chopra H. Khan Z. Contreras J. Wang H. Sedrak A. Zhu Y. (2018). Activation of P53 and Destabilization of Androgen Receptor by Combinatorial Inhibition of MDM2 and MDMX in Prostate Cancer Cells. Oncotarget 9 (5), 6270–6281. 10.18632/oncotarget.23569 29464071 PMC5814211

[B7] Ciccarese C. Massari F. Iacovelli R. Fiorentino M. Montironi R. Di Nunno V. (2017). Prostate Cancer Heterogeneity: Discovering Novel Molecular Targets for Therapy. Cancer Treat. Rev. 54, 68–73. 10.1016/j.ctrv.2017.02.001 28231559

[B8] Cronauer M. V. Schulz W. A. Burchardt T. Ackermann R. Burchardt M. (2004). Inhibition of P53 Function Diminishes Androgen Receptor-Mediated Signaling in Prostate Cancer Cell Lines. Oncogene 23 (20), 3541–3549. 10.1038/sj.onc.1207346 15077179

[B9] de Bono J. S. Logothetis C. J. Molina A. Fizazi K. North S. Chu L. (2011). Abiraterone and Increased Survival in Metastatic Prostate Cancer. N. Engl. J. Med. 364 (21), 1995–2005. 10.1056/NEJMoa1014618 21612468 PMC3471149

[B10] De Laere B. Oeyen S. Mayrhofer M. Whitington T. van Dam P. J. Van Oyen P. (2019). TP53 Outperforms Other Androgen Receptor Biomarkers to Predict Abiraterone or Enzalutamide Outcome in Metastatic Castration-Resistant Prostate Cancer. Clin. Cancer Res. 25 (6), 1766–1773. 10.1158/1078-0432.CCR-18-1943 30209161 PMC6330086

[B11] Ding Q. Zhang Z. Liu J. J. Jiang N. Zhang J. Ross T. M. (2013). Discovery of RG7388, a Potent and Selective P53-MDM2 Inhibitor in Clinical Development. J. Med. Chem. 56 (14), 5979–5983. 10.1021/jm400487c 23808545

[B12] Duffy M. J. Synnott N. C. O'Grady S. (2022). Targeting P53 for the Treatment of Cancer. Semin. Cancer Biol. 79, 58–67. 10.1016/j.semcancer.2020.07.005 32741700

[B13] Fang Y. Liao G. Yu B. (2020). Small-molecule MDM2/X Inhibitors and PROTAC Degraders for Cancer Therapy: Advances and Perspectives. Acta Pharm. Sin B 10 (7), 1253–1278. 10.1016/j.apsb.2020.01.003 32874827 PMC7452049

[B14] Feng F. Y. Zhang Y. Kothari V. Evans J. R. Jackson W. C. Chen W. (2016). MDM2 Inhibition Sensitizes Prostate Cancer Cells to Androgen Ablation and Radiotherapy in a P53-dependent Manner. Neoplasia 18 (4), 213–222. 10.1016/j.neo.2016.01.006 27108384 PMC4840291

[B15] Fizazi K. Shore N. Tammela T. L. Ulys A. Vjaters E. Polyakov S. (2020). Nonmetastatic, Castration-Resistant Prostate Cancer and Survival with Darolutamide. N. Engl. J. Med. 383 (11), 1040–1049. 10.1056/NEJMoa2001342 32905676

[B16] Ge R. Wang Z. Montironi R. Jiang Z. Cheng M. Santoni M. (2020). Epigenetic Modulations and Lineage Plasticity in Advanced Prostate Cancer. Ann. Oncol. 31 (4), 470–479. 10.1016/j.annonc.2020.02.002 32139297

[B17] Harris W. P. Mostaghel E. A. Nelson P. S. Montgomery B. (2009). Androgen Deprivation Therapy: Progress in Understanding Mechanisms of Resistance and Optimizing Androgen Depletion. Nat. Clin. Pract. Urol. 6 (2), 76–85. 10.1038/ncpuro1296 19198621 PMC2981403

[B18] Hussain M. Mateo J. Fizazi K. Saad F. Shore N. Sandhu S. (2020). Survival with Olaparib in Metastatic Castration-Resistant Prostate Cancer. N. Engl. J. Med. 383 (22), 2345–2357. 10.1056/NEJMoa2022485 32955174

[B19] Isaacsson Velho P. Fu W. Wang H. Mirkheshti N. Qazi F. Lima F. A. S. (2020). Wnt-pathway Activating Mutations Are Associated with Resistance to First-Line Abiraterone and Enzalutamide in Castration-Resistant Prostate Cancer. Eur. Urol. 77 (1), 14–21. 10.1016/j.eururo.2019.05.032 31176623 PMC6893106

[B20] Khor L. Y. Bae K. Paulus R. Al-Saleem T. Hammond M. E. Grignon D. J. (2009). MDM2 and Ki-67 Predict for Distant Metastasis and Mortality in Men Treated with Radiotherapy and Androgen Deprivation for Prostate Cancer: RTOG 92-02. J. Clin. Oncol. 27 (19), 3177–3184. 10.1200/JCO.2008.19.8267 19470936 PMC2716939

[B21] Konopleva M. Martinelli G. Daver N. Papayannidis C. Wei A. Higgins B. (2020). MDM2 Inhibition: an Important Step Forward in Cancer Therapy. Leukemia 34 (11), 2858–2874. 10.1038/s41375-020-0949-z 32651541

[B22] Ku S. Y. Rosario S. Wang Y. Mu P. Seshadri M. Goodrich Z. W. (2017). Rb1 and Trp53 Cooperate to Suppress Prostate Cancer Lineage Plasticity, Metastasis, and Antiandrogen Resistance. Science 355 (6320), 78–83. 10.1126/science.aah4199 28059767 PMC5367887

[B23] Levine A. J. (2020). p53: 800 Million Years of Evolution and 40 Years of Discovery. Nat. Rev. Cancer 20 (8), 471–480. 10.1038/s41568-020-0262-1 32404993

[B24] Li Y. Yang J. Aguilar A. McEachern D. Przybranowski S. Liu L. (2019). Discovery of MD-224 as a First-In-Class, Highly Potent, and Efficacious Proteolysis Targeting Chimera Murine Double Minute 2 Degrader Capable of Achieving Complete and Durable Tumor Regression. J. Med. Chem. 62 (2), 448–466. 10.1021/acs.jmedchem.8b00909 30525597 PMC6545112

[B25] Lin H. K. Wang L. Hu Y. C. Altuwaijri S. Chang C. (2002). Phosphorylation-dependent Ubiquitylation and Degradation of Androgen Receptor by Akt Require Mdm2 E3 Ligase. EMBO J. 21 (15), 4037–4048. 10.1093/emboj/cdf406 12145204 PMC126152

[B26] Logan I. R. McClurg U. L. Jones D. L. O'Neill D. J. Shaheen F. S. Lunec J. (2016). Nutlin-3 Inhibits Androgen Receptor-Driven C-FLIP Expression, Resulting in Apoptosis of Prostate Cancer Cells. Oncotarget 7 (46), 74724–74733. 10.18632/oncotarget.12542 27729622 PMC5342697

[B27] Maughan B. L. Guedes L. B. Boucher K. Rajoria G. Liu Z. Klimek S. (2018). p53 Status in the Primary Tumor Predicts Efficacy of Subsequent Abiraterone and Enzalutamide in Castration-Resistant Prostate Cancer. Prostate Cancer Prostatic Dis. 21 (2), 260–268. 10.1038/s41391-017-0027-4 29302046

[B28] Mu P. Zhang Z. Benelli M. Karthaus W. R. Hoover E. Chen C. C. (2017). SOX2 Promotes Lineage Plasticity and Antiandrogen Resistance in TP53- and RB1-Deficient Prostate Cancer. Science 355 (6320), 84–88. 10.1126/science.aah4307 28059768 PMC5247742

[B29] Neeb A. Herranz N. Arce-Gallego S. Miranda S. Buroni L. Yuan W. (2021). Advanced Prostate Cancer with ATM Loss: PARP and ATR Inhibitors. Eur. Urol. 79 (2), 200–211. 10.1016/j.eururo.2020.10.029 33176972

[B30] Niu Y. Chang T. M. Yeh S. Ma W. L. Wang Y. Z. Chang C. (2010). Differential Androgen Receptor Signals in Different Cells Explain Why Androgen-Deprivation Therapy of Prostate Cancer Fails. Oncogene 29 (25), 3593–3604. 10.1038/onc.2010.121 20440270

[B31] Nyquist M. D. Corella A. Coleman I. De Sarkar N. Kaipainen A. Ha G. (2020). Combined TP53 and RB1 Loss Promotes Prostate Cancer Resistance to a Spectrum of Therapeutics and Confers Vulnerability to Replication Stress. Cell Rep 31 (8), 107669. 10.1016/j.celrep.2020.107669 32460015 PMC7453577

[B32] Penson D. F. Armstrong A. J. Concepcion R. Agarwal N. Olsson C. Karsh L. (2016). Enzalutamide versus Bicalutamide in Castration-Resistant Prostate Cancer: The STRIVE Trial. J. Clin. Oncol. 34 (18), 2098–2106. 10.1200/JCO.2015.64.9285 26811535

[B33] Shu L. Li Z. Gu C. Fishlock D. (2013). Synthesis of a Spiroindolinone Pyrrolidinecarboxamide MDM2 Antagonist. Org. Process. Res. Dev. 17 (2), 247–256. 10.1021/op3003213

[B34] Skalniak L. Surmiak E. Holak T. A. (2019). A Therapeutic Patent Overview of MDM2/X-Targeted Therapies (2014-2018). Expert Opin. Ther. Pat 29 (3), 151–170. 10.1080/13543776.2019.1582645 30822185

[B35] Sung H. Ferlay J. Siegel R. L. Laversanne M. Soerjomataram I. Jemal A. (2021). Global Cancer Statistics 2020: GLOBOCAN Estimates of Incidence and Mortality Worldwide for 36 Cancers in 185 Countries. CA Cancer J. Clin. 71 (3), 209–249. 10.3322/caac.21660 33538338

[B36] Tovar C. Higgins B. Kolinsky K. Xia M. Packman K. Heimbrook D. C. (2011). MDM2 Antagonists Boost Antitumor Effect of Androgen Withdrawal: Implications for Therapy of Prostate Cancer. Mol. Cancer 10, 49. 10.1186/1476-4598-10-49 21539745 PMC3094321

[B37] Venkatesan T. Alaseem A. Chinnaiyan A. Dhandayuthapani S. Kanagasabai T. Alhazzani K. (2018). MDM2 Overexpression Modulates the Angiogenesis-Related Gene Expression Profile of Prostate Cancer Cells. Cells 7 (5). 10.3390/cells7050041 PMC5981265 29748481

[B38] Wade M. Li Y. C. Wahl G. M. (2013). MDM2, MDMX and P53 in Oncogenesis and Cancer Therapy. Nat. Rev. Cancer 13 (2), 83–96. 10.1038/nrc3430 23303139 PMC4161369

[B39] Watson P. A. Arora V. K. Sawyers C. L. (2015). Emerging Mechanisms of Resistance to Androgen Receptor Inhibitors in Prostate Cancer. Nat. Rev. Cancer 15 (12), 701–711. 10.1038/nrc4016 26563462 PMC4771416

[B40] Wyatt A. W. Azad A. A. Volik S. V. Annala M. Beja K. McConeghy B. (2016). Genomic Alterations in Cell-free DNA and Enzalutamide Resistance in Castration-Resistant Prostate Cancer. JAMA Oncol. 2 (12), 1598–1606. 10.1001/jamaoncol.2016.0494 27148695 PMC5097690

[B41] Yi H. Yan X. Luo Q. Yuan L. Li B. Pan W. (2018). A Novel Small Molecule Inhibitor of MDM2-P53 (APG-115) Enhances Radiosensitivity of Gastric Adenocarcinoma. J. Exp. Clin. Cancer Res. 37 (1), 97. 10.1186/s13046-018-0765-8 29716622 PMC5930807

[B42] Zhao Y. Liu L. Sun W. Lu J. McEachern D. Li X. (2013). Diastereomeric Spirooxindoles as Highly Potent and Efficacious MDM2 Inhibitors. J. Am. Chem. Soc. 135 (19), 7223–7234. 10.1021/ja3125417 23641733 PMC3806051

