# Supplementary material for: Novel MDM2 Inhibitor XR-2 Exerts Potent Anti-Tumor Efficacy and Overcomes Enzalutamide Resistance in Prostate Cancer
Source: Front Pharmacol. 2022 Apr 25;13:871259. doi: 10.3389/fphar.2022.871259 (PMC9081362; doi:10.3389/fphar.2022.871259)
Supplement: Supplementary file 1 [file Table1.DOC]

**Table S1. Primer sequences for qRT-PCR.**

| Segments | Primer sequences |
| --- | --- |

GAPDH up: 5’-GGTATCGTGGAAGGACTCATGAC-3’-3’

down: 5’-ATGCCAGTGAGCTTCCCGTTCAG-3’-3’

p53 up: 5’-CCTCAGCATCTTATCCGAGTGG-3’

down: 5’-TGGATGGTGGTACAGTCAGAGC-3’

MDM2 up: 5’-TGTTTGGCGTGCCAAGCTTCTC-3’

down: 5’-CACAGATGTACCTGAGTCCGATG-3’

P21 up: 5’-ATGAAATTCACCCCCTTTCC-3’

down: 5’-CCCTAGGCTGTGCTCACTTC-3’

PUMA up: 5’-GACGACCTCAACGCACAGTA-3’

down: 5’- AGGAGTCCCATGATGAGATTGT-3’

Bax up: 5’-TCAGGATGCGTCCACCAAGAAG-3’

down: 5’-TCAGGATGCGTCCACCAAGAAG-3’

GADD45A up: 5’- CTGGAGGAAGTGCTCAGCAAAG-3’

down: 5’-AGAGCCACATCTCTGTCGTCGT-3’
