# Supplementary material for: Novel MDM2 Inhibitor XR-2 Exerts Potent Anti-Tumor Efficacy and Overcomes Enzalutamide Resistance in Prostate Cancer
Source: Front Pharmacol. 2022 Apr 25;13:871259. doi: 10.3389/fphar.2022.871259 (PMC9081362; doi:10.3389/fphar.2022.871259)
Supplement: Supplementary file 2 [file Table2.DOC]

**Table S2. Primer sequences for p53 siRNA**

| Segments | Primer sequences |
| --- | --- |

p53-siRNA-1 sense: 5’-GCUUCGAGAUGUUCCGAGATT-3’

antisense: 5’-UCUCGGAACAUCUCGAAGCTT-3’

p53-siRNA-2 sense: 5’- GCAUCUUAUCCGAGUGGAATT-3’

antisense: 5’- UUCCACUCGGAUAAGAUGCTT-3’
